# HMGB1 mediates hyperglycaemia-induced cardiomyocyte apoptosis *via* ERK/Ets-1 signalling pathway

**DOI:** 10.1111/jcmm.12399

**Published:** 2014-09-11

**Authors:** Wen-Ke Wang, Qing-Hua Lu, Jia-Ning Zhang, Ben Wang, Xiang-Juan Liu, Feng-Shuang An, Wei-Dong Qin, Xue-Ying Chen, Wen-Qian Dong, Cheng Zhang, Yun Zhang, Ming-Xiang Zhang

**Affiliations:** a The Key Laboratory of Cardiovascular Remodeling and Function Research, Chinese Ministry of Education and Chinese Ministry of Public Health, Qilu Hospital of Shandong UniversityJinan, Shandong, China; b Department of Cardiology, The Second Hospital of Shandong UniversityJinan, China; c School of Foreign Languages and Literature, Shandong UniversityJinan, Shandong, China; d Department of Hepatobiliary Surgery, Qilu Hospital of Shandong UniversityJinan, Shandong, China

**Keywords:** high glucose, cardiomyocyte, apoptosis, diabetes, HMGB1, Ets-1

## Abstract

Apoptosis is a key event involved in diabetic cardiomyopathy. The expression of high mobility group box 1 protein (HMGB1) is up-regulated in diabetic mice. However, the molecular mechanism of high glucose (HG)-induced cardiomyocyte apoptosis remains obscure. We aimed to determine the role of HMGB1 in HG-induced apoptosis of cardiomyocytes. Treating neonatal primary cardiomyocytes with HG increased cell apoptosis, which was accompanied by elevated levels of HMGB1. Inhibition of HMGB1 by short-hairpin RNA significantly decreased HG-induced cell apoptosis by reducing caspase-3 activation and ratio of Bcl2-associated X protein to B-cell lymphoma/leukemia-2 (bax/bcl-2). Furthermore, HG activated E26 transformation-specific sequence-1 (Ets-1), and HMGB1 inhibition attenuated HG-induced activation of Ets-1 *via* extracellular signal-regulated kinase 1/2 (ERK1/2) signalling. In addition, inhibition of Ets-1 significantly decreased HG-induced cardiomyocyte apoptosis. Similar results were observed in streptozotocin-treated diabetic mice. Inhibition of HMGB1 by short-hairpin RNA markedly decreased myocardial cell apoptosis and activation of ERK and Ets-1 in diabetic mice. In conclusion, inhibition of HMGB1 may protect against hyperglycaemia-induced cardiomyocyte apoptosis by down-regulating ERK-dependent activation of Ets-1.

## Introduction

Diabetes mellitus is an increasing worldwide epidemic disease 1. Hyperglycaemia is the major feature of diabetes mellitus and can induce organ damage such as cardiovascular disease, the most frequent cause of death in the diabetic population 2. Heart failure in diabetes, which occurs independent of changes in blood pressure and coronary artery disease, is called diabetic cardiomyopathy 3. Increased cardiomyocyte apoptosis has been detected in hearts of diabetic patients and animal models of diabetes, and the loss of cardiomyocytes has been implicated in the development of diabetic cardiomyopathy 4. Diabetic patients with dilated cardiomyopathy showed significantly more apoptotic cardiomyocytes than patients without diabetes 5.

Multiple mechanisms for hyperglycaemia-induced cardiomyopathy have been proposed 6. The process of diabetic cardiomyopathy consists of a series of sequential and interrelated steps, including myocardial apoptosis, hypertrophy and fibrosis. Cardiomyocyte apoptosis is the keystone in the process 7. Cardiomyocytes release cytokines and produce reactive oxygen species (ROS) under high glucose (HG) conditions or hyperglycaemia, which can trigger cell death signalling cascades 8. However, little is known about the molecular mechanisms that regulate cardiomyocyte apoptosis under hyperglycaemia.

High mobility group box 1 protein (HMGB1) is a non-chromosomal nuclear protein that regulates gene transcription and maintains the nucleosome structure; it can be released from necrotic or activated immune cells 9. HMGB1 can be secreted by viable cardiomyocytes under certain pathological conditions 10. HMGB1 also serves as a molecular switch and regulates the activation of mitogen-activated protein kinases (MAPKs) 11. Toll-like receptor 4 (TLR4) is a commonly expressed receptor in cardiomyocytes, and could mediate the inflammatory response and apoptosis 12. Released HMGB1, together with its receptor TLR4, activates a downstream signal pathway for a significant role in cardiac apoptosis 13,14.

E26 transformation-specific (Ets) transcription family members share a highly conserved DNA-binding domain and are involved in regulating multiple biological processes including oncogenic transformation, angiogenesis, differentiation and apoptosis 14–17. Ets-1 is a member of this family. Ets transcription factors are downstream of extracellular signal-regulated kinase 1/2 (ERK1/2), which is important for activation of Ets-1 18. However, the role of Ets-1 as a regulator of cardiomyocyte apoptosis *in vivo* has not been defined.

Recently, we verified that HMGB1 promoted diabetes-induced myocardial fibrosis and heart dysfunction 19. Thus, we hypothesized that increased HMGB1 level may facilitate HG or hyperglycaemia-induced cardiomyocyte apoptosis. Here, we investigated the potential role and underlying mechanism of HMGB1 involved in HG-induced neonatal cardiomyocyte apoptosis *in vitro* and *in vivo*.

## Materials and methods

### Neonatal primary cardiomyocyte culture

Primary cardiomyocytes were isolated from 1- to 2-day-old rat ventricular tissues. Ventricular cells were dispersed by a digestion solution at 37°C, then resuspended in DMEM containing 10% foetal bovine serum, 5.5 mmol/l glucose and 2 mmol/l glutamine in a humidified incubator with 5% CO_2_ and 95% air. After 3–4 days, cells were incubated in serum-free essential medium overnight before treatment with HG (33 mmol/l), high mannose [osmotic control (OC); 5.5 mmol/l glucose + 27.5 mmol/l mannose] or normal glucose (NG; 5.5 mmol/l).

### HMGB1 knockdown in cardiomyocytes

Cardiomyocytes were cultured in 6-well culture plates and infected with a lentivirus vector containing HMGB1 short-hairpin RNA (shRNA) at multiplicity of infection of 10 for 24 hrs. The target sequence for HMGB1 was 5′-GGCTCGTTATGAAAGAGAAAT-3′ and negative control sequence 5′-GTTCTCCGAACGTGTCACGT-3′. Ets-1 was silenced with a small interfering RNA (siRNA) with the sequence 5′-GCUACCUUCAGUGGUUUCATT-3′ (Gene Pharma, Shanghai, China) by use of Lipofectamine 2000 (Invitrogen, Carlsbad, CA, USA); cells were incubated at 37°C for 48 hrs, then cultured with HG (33 mmol/l) for 24 hrs.

### Western blot analysis

Cardiomyocytes and mouse heart tissues were lysed by radioimmunoprecipitation assay. Equal amounts of protein from different experimental groups were separated on 10% SDS-PAGE and transferred to PVDF membranes (Millipore, Eschborn, Germany), which were blocked for 1 hr with 5% non-fat milk in TBST at room temperature, then incubated overnight at 4°C with primary antibodies against HMGB1 (Abcam, Cambridge, MA), phospho-Ets-1 (at Thr38; Assay Biotech), total Ets-1, cleaved caspase-3, Bax, Bcl-2, phospho-ERK1/2, total ERK1/2 or β-actin (all Cell Signaling Technology), then horseradish peroxidase-conjugated secondary antibody for 1 hr at room temperature. Membranes were visualized by a chemiluminescence kit (Millipore, Billerica, MA, USA).

### TUNEL staining

DNA fragmentation was examined by use of a commercial kit for detecting apoptosis (ApopTag Plus Peroxidase, Chemicon, Temecula, CA, USA). After deparaffinization and hydration, mouse heart tissue sections underwent enzymatic digestion with 20 μg/ml proteinase K for 5 min. and were washed with PBS. Then, equilibration buffer was applied for 5 min. and sections were incubated with working strength terminal deoxynucleotidyl transferase enzyme at 37°C for 1 hr in a humidified chamber, washed with PBS and incubated with anti-digoxigenin conjugate and peroxidase substrate to detect signs of apoptosis, stained brown. Counterstain was carried out with 0.5% methyl green.

### Confocal microscopy

Neonatal cardiomyocytes were seeded on glass coverslips in 24-well culture plates. For phospho-Ets-1 (Thr38) analysis, cardiomyocytes were treated with HG after transfection of shRNA HMGB1 or U0126, washed with PBS, and then fixed with 4% paraformaldehyde for 20 min. at room temperature. Cells were washed three times with PBS, blocked in 5% goat serum for 1 hr, and incubated with primary antibody for phospho-Ets-1 (Thr38) in PBS with 0.1% Triton X-100 in a humidified chamber overnight at 4°C, then washed with PBS before incubation with secondary antibody (1:500 dilution; Jackson Laboratories, West Grove, Pennsylvania) for 30 min. at 37°C. Cells were washed three times with PBS and coverslips were sealed by use of a drop of Prolong Gold antifade reagent with DAPI (Invitrogen). Images were acquired by laser scanning confocal microscopy (LSM710; Zeiss, Jena, Germany) and analysed by use of Image-Pro Plus 6.0.

### Mouse models

C57BL/6J male mice were randomly allocated to two groups for treatment: diabetes and non-diabetes. At 8 weeks of age, diabetic mice received five consecutive daily intraperitoneally injections of streptozotocin (STZ, 60 mg/kg bodyweight, in 0.1 mol/l citrate buffer, ph 4.5; Sigma-Aldrich, St Louis, MO, USA) to induce diabetes and non-diabetic mice received injections of citrate buffer vehicle of equivalent volume. Blood glucose was measured from the tail vein by use of a glucometer (ACCU-CHEK Advantage; Roche, Indianapolis, Indiana, USA). Mice with blood glucose levels ≥16.7 mmol/l were considered diabetic. To knock down HMGB1 expression, an shRNA against mouse HMGB1 was transfected into mouse hearts and a scramble shRNA was employed as a control. After induction of diabetes for 8 weeks, an amount of 1 × 10^7^ UT/30 μl of lentivector with HMGB1 shRNA or the same volume of lenti-vehicle was injected into three sites of the left ventricle under thoracotomy. After 12 weeks, mice were killed. All experiments conformed to the Guide for the Care and Use of Laboratory Animal published by the U.S. National Institutes of Health and Shandong University. The study protocol was approved by the Institutional Ethics Committee of Shandong University.

### Immunohistochemistry

Paraffinized mouse heart sections 5-μm thick underwent routine deparaffinization and rehydration and were boiled in sodium citrate buffer solution (pH 6.0) at 95°C for 15 min. Sections were incubated overnight at 4°C with primary antibodies against rabbit HMGB1 or phospho-Ets-1 (Thr38), then with horseradish peroxidase-conjugated anti-rabbit antibody. HMGB1- or phospho-Ets-1-treated samples were stained with 3,3-diaminobenzidine. Normal rabbit IgG was substituted for primary antibody as the negative control.

### Statistical analysis

Data are presented as mean ± SD of three independent experiments and analysed by Student’s *t*-test for two groups and one-way anova for multiple groups by use of SPSS 18.0 (SPSS Inc., Chicago, IL, USA). Differences were considered statistically significant at *P* < 0.05.

## Results

### HG induced apoptosis of neonatal primary cardiomyocytes

**Figure 1:**
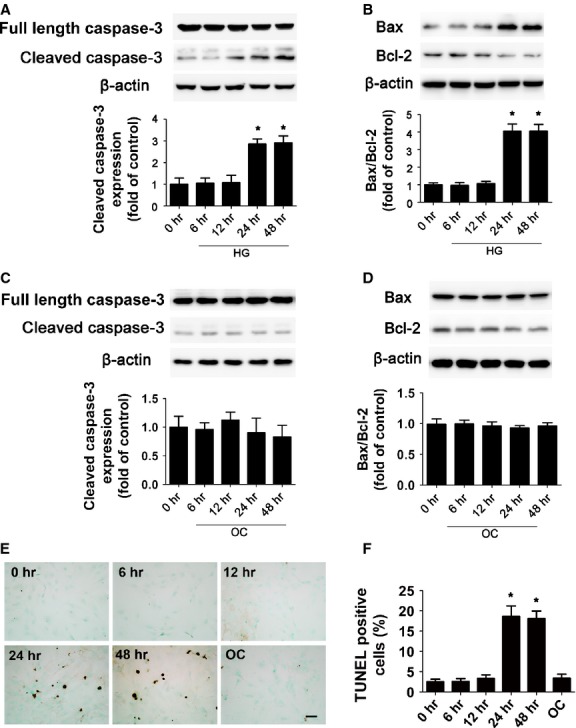
High glucose induced cardiomyocyte apoptosis. The protein expression of cleaved caspase-3 (**A**), Bax and Bcl-2 (**B**) with high glucose (HG; 33 mmol/l) treatment was determined by Western blot. (**C** and **D**) The protein expression of cleaved caspase-3 (**C**), Bax and Bcl-2 (**D**) with OC (5.5 mmol/l glucose plus 27.5 mmol/l mannose) treatment was determined by Western blot. Quantitative data are expressed as fold of control (normal glucose: 5.5 mmol/l glucose). (**E** and **F**) Apoptosis rate measured by TUNEL assay at different times after HG treatment (scale bar: 20 μm). Data are mean ± SD of three independent experiments. HG: high glucose. NG: normal glucose. OC: osmotic control. *P < 0.05 compared with time 0.

An HG dose (33 mmol/l) was commonly used in previous studies to investigate the effect of HG on apoptosis of cardiomyocytes 8,20–22. Moreover, in our preliminary study, we used NG (5.5 mmol/l), medium glucose (16.7 mmol/l) and HG (33 mmol/l) to investigate the effect of HG on inducing cardiomyocyte apoptosis and found that 33 mmol/l HG treatment induced a marked increase in the apoptosis of cardiomyocyte (data not shown). So we employed 33 mmol/l HG in our experiment. Neonatal primary cardiomyocytes were treated with HG for different times. The expression of cleaved caspase-3 was higher in cardiomyocytes with HG than NG treatment at 24 and 48 hrs (both *P* < 0.05; Fig. 1A). Similarly, the ratio of Bax to Bcl-2 was increased with HG treatment at 24 and 48 hrs (both *P* < 0.05; Fig. 1B). The apoptosis rate detected by TUNEL assay demonstrated that HG treatment increased the per cent of apoptotic cardiomyocytes at 24 and 48 hrs (both *P* < 0.05; Fig. 1E and F). Levels of cleaved caspase-3, Bax/Bcl-2 ratio and TUNEL-positive cells did not differ over time with isotonic mannose treatment (OC: 5.5 mmol/l glucose+ 27.5 mmol/l mannose) as compared with NG (Fig. 1C and D). The effect of HG on cardiomyocyte apoptosis was similar at 24 and 48 hrs, so we chose the 24 hrs time-point for further study.

### HMGB1 was required for HG-induced cardiomyocyte apoptosis

**Figure 2:**
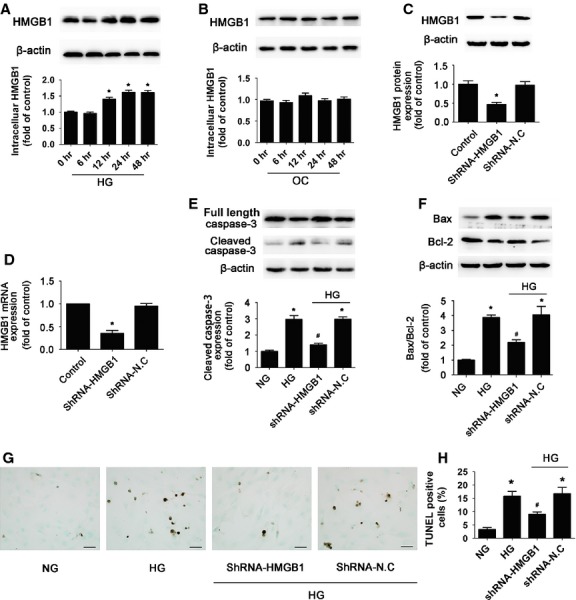
High glucose treatment increased cardiomyocyte intracellular HMGB1 and inhibition of HMGB1 reduced high glucose-induced apoptosis. Neonatal primary cardiomyocytes were treated with HG (33 mmol/l glucose) or OC (5.5 mmol/l glucose + 27.5 mmol/l mannose) for different times. (**A**) Western blot analysis of the protein level of intracelluar HMGB1 with HG treatment. (**B**) Western blot analysis of the protein level of intracelluar HMGB1 with OC treatment. Quantitative data are expressed as fold of control (NG: 5.5 mmol/l glucose). Cardiomyocytes were transfected with HMGB1-shRNA or negative control shRNA (shRNA-N.C) for 24 hrs, then incubated with HG. Western blot (**C**) and RT-PCR (**D**) analysis of the silencing efficacy of HMGB1-shRNA. Western blot analysis of the protein levels of cleaved caspase-3 (**E**), Bax and Bcl-2 (**F**) with HMGB1 inhibition. (**G** and **K**) The apoptosis rate was determined by TUNEL assay (scale bar: 20 μm). Data are mean ± SD of three independent experiments. HG: high glucose. NG: normal glucose. OC: osmotic control. *P < 0.05 compared with control or NG; ^#^*P* < 0.05 compared with HG or HG+shN.C.

The cytokine HMGB1 is involved in sepsis-induced myocyte apoptosis 23. To assess whether HMGB1 plays a role in HG-induced cardiomyocyte apoptosis, we evaluated HMGB1 expression in HG-treated cardiomyocytes for various times. Cardiomyocyte HMGB1 expression started to increase at 12 hrs and peaked at 24 hrs with HG as compared with NG treatment (*P* < 0.05; Fig. 2A). These effects were not observed with high osmolarity (OC) treatment (Fig. 2B). To determine whether HMGB1 contributed to HG-induced apoptosis of cardiomyocytes, we transfected cardiomyocytes with HMGB1-specific shRNA for 24 hrs, and then incubated them with HG. The transfection efficacy of specific shRNA reached 90% (data not shown) and the protein and mRNA levels of HMGB1 were significantly decreased after transfection as compared with negative control shRNA treatment (*P* < 0.05; Fig. 2C and D), which suggested successful knock-down. As compared with HG alone, HMGB1 inhibition with HG significantly reduced cardiomyocyte apoptosis. HG increased the level of cleaved caspase-3 (*P* < 0.05; Fig. 2E) and Bax/Bcl-2 ratio (*P* < 0.05; Fig. 2F) as well as number of TUNEL-positive cells (*P* < 0.05; Fig. 2G and H), whereas inhibition of HMGB1 attenuated the HG-induced apoptotic effect (*P* < 0.05; Fig. 2E–H). Thus, HMGB1 was required for HG-induced apoptosis.

### HMGB1 was essential for HG-induced activation of Ets-1

**Figure 3:**
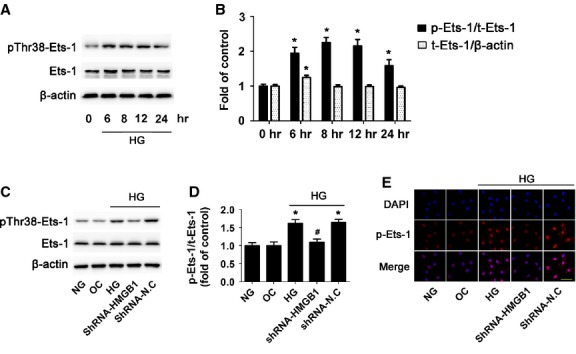
High glucose treatment increased phosphorylated Ets-1 and HMGB1 inhibition decreased high glucose-induced activation of Ets-1. Cardiomyocytes were cultured in HG medium for different times. (**A**) Western blot analysis of protein level of phospho-Ets-1 (Thr38) and total Ets-1. (**B**) Quantification of phospho-Ets-1/Ets-1 and Ets-1 expression was shown. Data are mean ± SD of three independent experiments. *P < 0.05 compared with time 0. (**C** and **D**) Western blot analysis of level of pThr38-Ets-1 and total Ets-1 with HMGB1 inhibition. (**E**) Confocal microscopy of pThr38-Ets-1 (scale bar: 50 μm). Data are mean ± SD of three independent experiments. HG: high glucose. NG: normal glucose. OC: osmotic control. *P < 0.05 compared with control or NG; ^#^*P* < 0.05 compared with HG or HG+shN.C.

Given that Ets-1 is a key transcription factor that regulates growth and apoptosis 24, we wondered whether HG-induced cardiomyocyte apoptosis was associated with Ets-1 activation. Our results showed that after HG treatment for 6 hrs, the protein level of total Ets-1 protein expression was slightly increased and phospho-Ets-1 (Thr38) was significantly elevated at 6 hrs up to 24 hrs (*P* < 0.05; Fig. 3A and B). To investigate whether HMGB1 regulates HG-induced activation of Ets-1, cardiomyocytes were transfected with HMGB1 shRNA, and phospho-Ets-1 level was assessed. Our results demonstrated that HG but not high osmolarity (OC) activated Ets-1 (HG *versus* NG or OC, *P* < 0.05) and accumulation of phosphorylated Ets-1 in the nucleus; inhibition of HMGB1 effectively reversed HG-increased level of phosphorylated Ets-1 (HG+shRNA-HMGB1 *versus* HG or HG+shN.C, *P* < 0.05) and its accumulation in the nucleus (Fig. 3C–E). Therefore, HMGB1 plays an essential role in HG-induced activation of Ets-1.

### Inhibition of Ets-1 reduced HG-induced cardiomyocytes apoptosis

**Figure 4:**
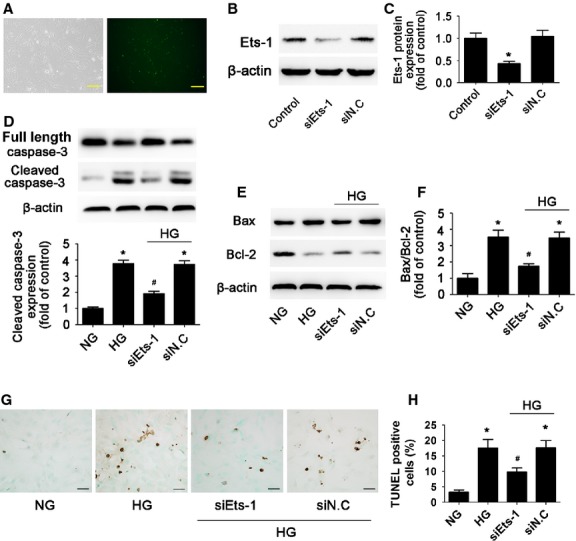
Inhibition of Ets-1 reduced high glucose-induced cardiomyocytes apoptosis. Cardiomyocytes were transfected with Ets-1 siRNA. (**A**) Transfection efficacy of siRNA (scale bar: 50 μm). (**B** and **C**) Efficacy of siRNA-Ets-1 was determined by Western blot. Western blot analysis of the protein levels of cleaved caspase-3 (**D**), Bax and Bcl-2 (**E** and **F**) with siRNA inhibition of Ets-1. (**G** and **H**) TUNEL assay of apoptosis rate of cardiomyocytes (scale bar: 20 μm). Data are mean ± SD of three independent experiments. HG: high glucose. NG: normal glucose. *P < 0.05 compared with control or NG; ^#^*P* < 0.05 compared with HG or HG+siN.C.

To further confirm whether Ets-1 activation was involved in HG-induced cardiomyocyte apoptosis, we used Ets-1 siRNA to knock-down its protein level and then treated cells with HG. The transfection efficacy of Ets-1 siRNA reached 90% (Fig. 4A). Compared with the control, Ets-1 expression was significantly reduced by siRNA (*P* < 0.05; Fig. 4B and C). HG stimulation markedly increased cardiomyocyte apoptosis, while siRNA inhibition of Ets-1 significantly reduced the activation of caspase-3 protein (*P* < 0.05; Fig. 4D), Bax/Bcl-2 ratio (*P* < 0.05; Fig. 4E and F) and cell apoptosis (*P* < 0.05; Fig. 4G and H) as compared with HG alone. Thus, HMGB1 inhibition reduced HG-induced cardiomyocyte apoptosis by inhibiting activation of Ets-1.

### ERK pathway was involved in HMGB1-mediated activation of Ets-1

**Figure 5:**
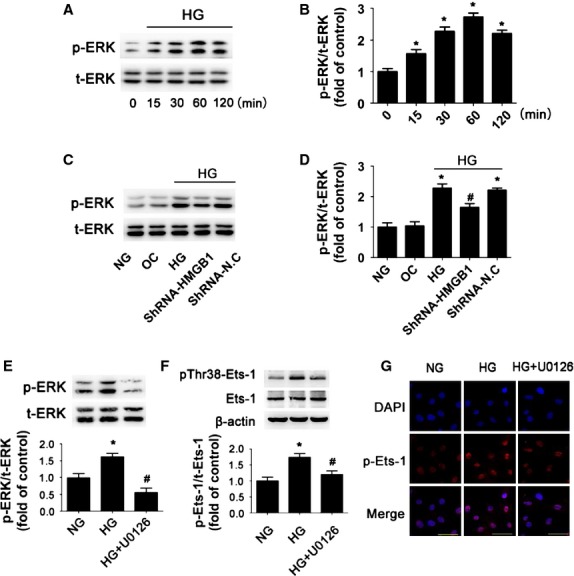
ERK pathway was involved in high glucose-induced activation of Ets-1. (**A** and **B**) Expression of total-ERK (t-ERK) and phospho-ERK (p-ERK) was detected by Western blot after HG stimulation for different times. (**C** and **D**) Expression of total-ERK (t-ERK) and phospho-ERK (p-ERK) was detected by Western blot after inhibition of HMGB1. (**E**) Expression of total-ERK (t-ERK) and phospho-ERK (p-ERK) was measured by Western blot after using U0126. (**F**) Expression of phosphorylated Ets-1 on Thr38 (pThr38-Ets-1) and Ets-1 was measured by Western blot. (**G**) Confocal microscopy of pThr38-Ets-1 with immunofluorescent staining (scale bar: 50 μm). Data are mean ± SD of three independent experiments. HG: high glucose. NG: normal glucose. OC: osmotic control. *P < 0.05 compared with NG; ^#^*P* < 0.05 compared with HG or HG+shN.C.

Previous data have shown that ERK is necessary for diabetic cardiomyopathy 25. It has shown that HG induced apoptosis by activation of ERK 26. It is also believed that phospho-ERK1/2 is considered a potent activator of Ets-1 27. We determined whether HG promoted Ets-1 activation through ERK signalling. Stimulation of cells with HG increased the phospho-ERK 1/2 level with a peak at 60 min. (*P* < 0.05; Fig. 5A and B). HG but not high OC increased the level of phospho-ERK1/2 in cardiomyocytes, whereas inhibition of HMGB1 with HMGB1-specific shRNA significantly attenuated HG-induced ERK1/2 activation in cardiomyocytes (*P* < 0.05; Fig. 5C and D). Furthermore, we examined whether HG enhanced Ets-1 activation *via* an ERK pathway. A specific ERK inhibitor (U0126) was applied 30 min. before HG exposure in cardiomyocytes. Pre-treating cardiomyocytes with U0126 prevented HG-increased phospho-ERK1/2 level (*P* < 0.05; Fig. 5E) and activation of Ets-1 (*P* < 0.05; Fig. 5F). HG significantly increased the nuclear level of phospho-Ets-1 (Thr38), which was reduced by U0126 (Fig. 5G). These observations supported that HMGB1 mediated Ets-1 activation *via* an ERK1/2 pathway under HG treatment.

### Diabetes-induced myocardial apoptosis was mediated by HMGB1 *in vivo*

**Figure 6:**
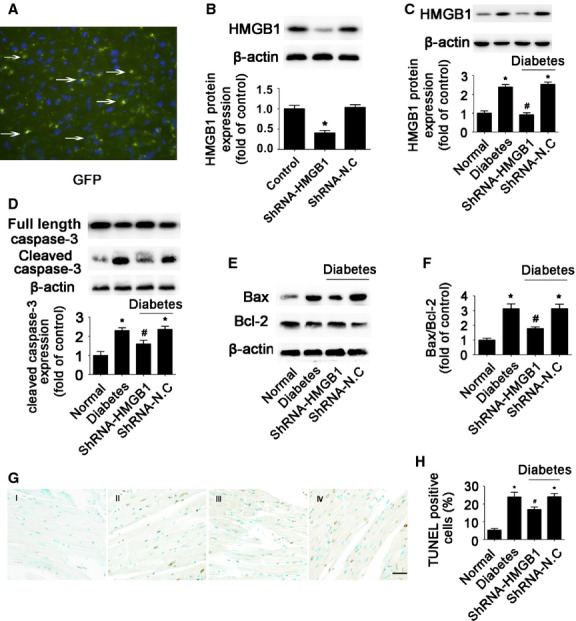
HMGB1 inhibition protected against diabetes-induced myocardial apoptosis *in vivo*. (**A**) Representative fluorescence microscopy of GFP-labelled scramble transfection efficiency in mouse myocardial tissue (original magnification ×400). (**B**) The efficacy of shRNA was determined by Western blot analysis. (**C**). Western blot analysis of HMGB1 in diabetes mice. The levels of cleaved caspase-3 (**D**), Bax and Bcl-2 (**E** and **F**) after HMGB1 inhibition were determined by Western blot. (**G** and **H**) TUNEL assay of cell apoptosis rate (scale bar: 20 μm). I: normal; II: diabetes; III: diabetes+shRNA-HMGB1; IV: diabetes+shRNA-N.C. Data are mean ± SD (*n* = 8). *P < 0.05 compared with normal; ^#^*P* < 0.05 compared with diabetes or diabetes+shRNA-N.C.

To examine the role of HMGB1 in myocardial apoptosis *in vivo*, we induced type 1 diabetes by STZ in mice. Our previous study revealed that hyperglycaemia significantly increased HMGB1 expression and secretion in STZ-treated hearts 19. To delineate the role of HMGB1 in diabetes-induced myocardial apoptosis, we silenced HMGB1 gene expression in myocardia of diabetic mice. The transfection efficacy of HMGB1 shRNA reached 60% (Fig. 6A). Transfection of shRNA-HMGB1 knocked down HMGB1 expression in normal mice (*P* < 0.05; Fig. 6B) and diminished its elevation in diabetic mice (Fig. 6C). Meanwhile, hyperglycaemia significantly increased myocardial caspase-3 activity (*P* < 0.05; Fig. 6D) and ratio of Bax/Bcl-2 (*P* < 0.05; Fig. 6E and F). In addition, the proportion of TUNEL-positive apoptotic cells was significantly increased in diabetic hearts (*P* < 0.05; Fig. 6G and H). HMGB1 inhibition effectively ameliorated hyperglycaemia-activated caspase-3 (*P* < 0.05; Fig. 6D) and decreased Bax/Bcl-2 ratio (*P* < 0.05; Fig. 6E and F). In addition, HMGB1 inhibition decreased the proportion of TUNEL-positive cells in the diabetic mouse (*P* < 0.05; Fig. 6G and H). Therefore, HMGB1 is involved in hyperglycaemia-induced myocardial apoptosis in diabetic mice.

### Knock-down of HMGB1 gene prevented ERK and Ets-1 activation in the diabetic mouse heart

**Figure 7:**
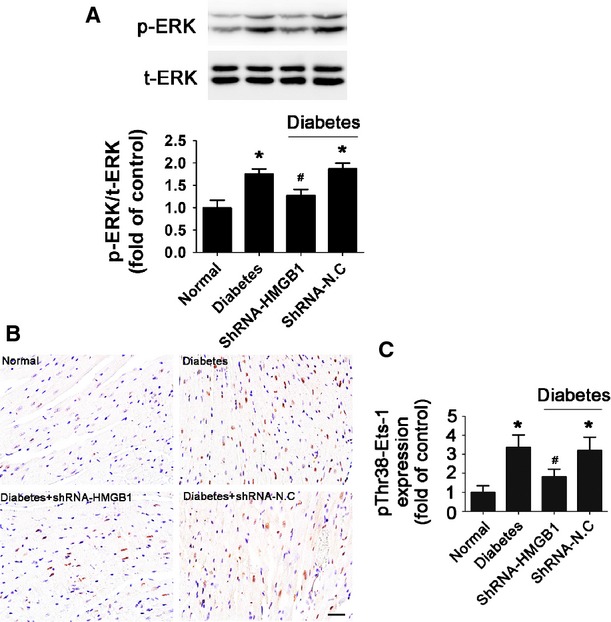
Inhibition of HMGB1 reduced hyperglycaemia-induced phosphorylation of ERK and Ets-1 *in vivo*. (**A**) Western blot analysis of the expression of total-ERK (t-ERK) and phospho-ERK (p-ERK) in mice. (**B** and **C**) Immunohistochemical staining and quantification of pThr38-Ets-1 (scale bar: 20 μm). Data are mean ± SD (*n* = 8). *P < 0.05 compared with normal; ^#^*P* < 0.05 compared with diabetes or diabetes+shRNA-N.C.

We investigated the activation of ERK and Ets-1 in hearts. Hyperglycaemia increased the phospho-ERK level, and inhibition of HMGB1 by shRNA significantly reduced hyperglycaemia-induced ERK phosphorylation (*P* < 0.05; Fig. 7A). Twelve weeks of hyperglycaemia significantly increased the level of phospho-Ets-1 as compared with citrate buffer-treated mice (*P* < 0.05; Fig. 7B and C). In contrast, silencing HMGB1 gene significantly decreased the level of phospho-Ets-1 in the diabetic myocardium (*P* < 0.05; Fig. 7B and C). These data suggested that HMGB1 mediated hyperglycaemia-induced activation of ERK and Ets-1 in diabetic mice.

## Discussion

Hyperglycaemia-induced cardiac apoptosis plays an important role in diabetic complications 4. Although HMGB1 has been implicated in hyperglycaemia-induced heart failure, the fundamental mechanism still remains unclear 28. Here, we focused on the potential role and mechanism of HMGB1 in HG-induced cardiomyocyte apoptosis. The major findings of our study are that: (*i*) HG increased the expression of HMGB1, which mediated the apoptosis of primary cardiomyocytes; (*ii*) HMGB1 mediates HG-induced ERK-dependent activation of Ets-1 and (*iii*) phosphorylation of Ets-1 led to apoptosis of cardiomyocytes with HG stimulation. We demonstrated that HMGB1 mediated apoptosis *via* the ERK-dependent activation of Ets-1 in both HG-treated cardiomyocytes and diabetic hearts. However, the effect of Ets-1 on diabetic myocardial dysfunction and myocardial cell death *in vivo* needs further investigation.

As a result of loss of terminally differentiated cardiomyocytes, apoptosis plays a key role in the pathogenesis of various cardiovascular diseases 29. Cardiomyocytes undergoing apoptosis have been identified in tissue samples from patients with diabetes and in animal models of diabetes 5. Apoptosis is a highly regulated programme of cell death. Anti-apoptotic members such as Bcl-2 are important in cell survival and protect cardiomyocytes against various stressors. Meanwhile, pro-apoptotic members such as Bax are essential for cell death mediated by the mitochondrial pathway 30. The balance between pro- and anti-apoptotic proteins determines cell survival or apoptosis with an apoptotic stimulus 31. In our study, we found that the expression of Bax was enhanced and that of Bcl-2 was reduced in cardiomyocytes under HG as compared with NG conditions. The continuous loss of cardiomyocytes triggers myocyte hypertrophy and fibrosis, but the molecular pathways involved in cardiomyocyte apoptosis induced by hyperglycaemia remain unclear. Many studies have demonstrated that inhibition of apoptosis is cardioprotective and can prevent the development of heart failure 32. Therefore, this process represents a potential target for therapeutic intervention to prevent hyperglycaemia-induced heart failure.

HMGB1 has been thought to play important roles in cardiomyocyte apoptosis induced by cardiac ischaemia-reperfusion and heart failure. HMGB1 induced Bax up-regulation and cytochrome c release in rat hearts after ischaemia-reperfusion injury 33. It has also been reported that HMGB1 and its receptor TLR4 contribute to cardiomyocyte apoptosis and enhance the inflammatory response to myocardial damage after ischaemia-reperfusion injury 34. In addition, HMGB1 is involved in doxorubicin-induced myocardial apoptosis 13. However, the role of HMGB1 in hyperglycaemia-induced apoptosis of cardiomyocytes has not been characterized. We previously found that HMGB1 expression and release were increased in cardiomyocytes exposed to HG and in the diabetic mouse heart. Inhibition of HMGB1 could attenuate diabetes-induced myocardial fibrosis and dysfunction 19. In the present study, we investigated the role and potential molecular pathway of HMGB1 involved in cardiomyocyte apoptosis. We observed that HMGB1 inhibition by shRNA reduced HG-induced caspase-3 activity and Bax/Bcl-2 ratio in both HG-stimulated cardiomyocytes and diabetic hearts. The apoptosis of cardiomyocytes in the HG or hyperglycaemia environment could be attenuated by knock-down of HMGB1 gene. It suggests that inhibition of HMGB1 may protect cardiomyocytes against apoptosis under hyperglycaemic conditions. In addition, some studies have demonstrated that conditional knockout HMGB1 in tissues or cells accelerate apoptosis and inflammation 35. Indeed, exogenous HMGB1 protein triggers cell survival or death in cancer cells, depending on its redox status 36. Reducible HMGB1 decreased cell death in cancer cells, whereas oxidized HMGB1 activated the mitochondrial apoptosis pathway. Oxidative stress occurs when the generation of ROS in a system exceeds its ability to neutralize and eliminate them. Because hyperglycaemia depletes natural antioxidants and facilitates the production of free radicals, increased oxidative stress occurs in diabetic patients 37,38. This suggests that hyperglycaemia may increase the proportion of oxidized HMGB1 and HMGB1 may promote cardiomyocyte apoptosis under HG or hyperglycaemia conditions. So, whether HMGB1 mediates cell survival or death depends on the underlying pathological condition.

We further investigated the potential mechanism of HMGB1 in HG-induced cardiomyocyte apoptosis. Ets-1 is a transcription factor, controlling cell growth, proliferation, differentiation and apoptosis 15. Previous studies showed that HG treatment increased the nuclear accumulation of Ets-1 protein and blocking the ERK pathway suppressed the nuclear accumulation of Ets-1 in podocytes 39. Overexpression of Ets-1 in human umbilical vein endothelial cells induced apoptosis under serum-deprived conditions 24. It is also a promoter of caspase-1 and can mediate colon cancer cell apoptosis 40. We found that HG treatment time-dependently up-regulated the phosphorylation of Ets-1 on Thr38. Moreover, the level of phospho-Ets-1 (Thr38) and its accumulation in the nucleus were significantly higher in neonatal primary cardiomyocytes exposed to HG rather than NG. Furthermore, inhibiting HMGB1 significantly reduced the HG-induced expression of phospho-Ets-1 (Thr38) in cardiomyocytes. *In vivo*, cardiac phospho-Ets-1 (Thr38) level was significantly increased in diabetic mice, HMGB1 inhibition reduced diabetes-induced activated Ets-1. This suggests that Ets-1 plays a crucial role in the process. We inhibited Ets-1 by siRNA to determine whether the activation of Ets-1 could increase cardiomyocyte apoptosis *in vitro*. As expected, Ets-1 inhibition significantly reduced HG-induced cell apoptosis. With hyperglycaemia, increased HMGB1 level could promote cardiac cell death *via* the nuclear transcription factor Ets-1.

Several potential pathways are considered to regulate diabetic cardiomyopathy, including MAPK cascade signalling 41. Although several studies reported that activation of ERK1/2 has anti-apoptotic roles in myocardial ischaemic/reperfusion injury 42, some studies support the pro-apoptotic role of ERK1/2 in cardiomyocytes. Previous studies showed that H2S prevented HG-induced cardiomyoblast (H9c2) apoptosis by inhibiting the activation of the ERK1/2 pathways 43. Silencing TRB3 reversed diabetes-induced myocardial remodelling by inhibiting phosphorylation of ERK1/2 41. Whether ERK1/2 activation has a pro- or anti-apoptotic effect on cardiomyocytes may depend on the different pathological condition. Other studies have shown that ERK1/2 pathways are involved in the HMGB1-mediated inflammatory response and apoptosis 44. As well, activated ERK1/2 is considered a potent activator of Ets-1 18. However, whether these signalling pathways are involved in HMGB1-mediated Ets-1 activation in cardiomyocytes exposed to HG was not known. We found that HG induced ERK phosphorylation, and inhibition of HMGB1 gene expression decreased the phosphorylation of ERK1/2 in cardiomyocytes exposed to HG. Moreover, the ERK inhibitor (U0126) robustly attenuated HG-induced Ets-1 activation. These finding are consistent with previous reports which showed that activated ERK could form a signalling complex with Ets-1 to facilitate its phosphorylation on Thr38, thus resulting in increased transcription activity. These data confirmed that HMGB1-mediated Ets-1 activation requires ERK1/2 activation.

In conclusion, hyperglycaemia is known to contribute to diabetic complications. HMGB1 plays a critical role in hyperglycaemia-induced cardiomyocyte apoptosis *via* ERK-dependent activation of Ets-1, thus leading to myocardial dysfunction in diabetes. Inhibition of HMGB1 may be a target for therapeutic treatment of diabetic cardiomyopathy.
